# Transorbital B-mode ultrasound for the assessment of posterior globe flattening in idiopathic intracranial hypertension: a pilot study

**DOI:** 10.1186/s13089-024-00388-z

**Published:** 2024-08-19

**Authors:** Theresia Knoche, Charlotte Pietrock, Konrad Neumann, Mirjam Rossel-Zemkouo, Leon Alexander Danyel

**Affiliations:** 1 Department of Neurology, Charité Universitätsmedizin Berlin – Campus Virchow Klinikum, Augustenburger Platz 1, 13353 Berlin, Germany; 2 Institute for Biometry and Clinical Epidemiology, Charité - Universitätsmedizin Berlin, Berlin, Germany; 3 Department of Ophthalmology, Charité Universitätsmedizin Berlin – Campus Virchow Klinikum, Berlin, Germany

**Keywords:** Posterior globe flattening, Sonography, Idiopathic intracranial hypertension, Transorbital ultrasound

## Abstract

**Background:**

Posterior globe flattening (PGF) is a specific neuroimaging sign in patients with idiopathic intracranial hypertension (IIH), but its detection is based on subjective qualitative neuroradiological assessment. This study sought to evaluate the utility of transorbital ultrasound to detect and quantify PGF in IIH patients using the *Posterior Globe Angle* (PGA).

**Methods:**

Consecutive IIH patients and healthy controls were enrolled in a prospective case-control study. Transorbital ultrasound was performed to assess the presence of PGF. For quantification of PGF, an angular measurement (PGA) was performed with the vertex centering the optic nerve at a predefined distance from the lamina cribrosa and angle legs tangentially aligned to the borders of the vitreous body. PGA measurements were compared between IIH patients and healthy controls. Additionally, the diagnostic accuracy of PGA measurements in detecting PGF was evaluated using ROC analysis.

**Results:**

Thirty-one IIH patients (37.3 ± 12.3 years, 29 female) and 28 controls (33.3 ± 11.8 years, 21 female) were compared. PGF was present in 39% of IIH patients and absent in the control group. PGA_3mm_ measurements significantly differed between IIH and controls (116.5° ± 5.5 vs. 111.7° ± 2.9; *p* < 0.001). A PGA_3mm_ cutoff of ≥ 118.5° distinguished IIH patients from controls with 100% specificity, while retaining a sensitivity of 37.5%.

**Conclusions:**

Transorbital ultrasound may be applied to detect and quantify PGF in IIH patients. Prospective, multicenter studies with extended cohorts and blinded design are needed to validate these preliminary findings and confirm the diagnostic utility of transorbital ultrasound for the assessment of PGF in IIH.

## Introduction

Idiopathic intracranial hypertension (IIH) is a rare headache syndrome mainly observed in obese women of reproductive age [1–3]. The condition is defined by the presence of an elevated intracranial pressure without identifiable source of hydrocephalus or cerebral mass lesions. Its underlying pathophysiology remains vague [4]. Principal symptoms are headaches, visual disturbance and diplopia [5]. In a retrospective review of 165 patients referred to the neuro-ophthalmology division of a tertiary care center due to suspected IIH, Fisayo et al. found a misdiagnosis rate of 39.5%, emphasizing the importance of a thorough diagnostic work-up and the application of current IIH diagnostic criteria [6]. According to the revised Friedman criteria a diagnosis of IIH can be established in patients with papilledema and elevated lumbar puncture opening pressure (≥ 25cmCSF) if alternate diagnoses have been excluded through adequate neurological examination, neuroimaging, and cerebrospinal fluid (CSF) analysis (Table 1) [7]. However, the Friedman criteria also allow for a diagnosis of “possible IIH” in patients without papilledema, if 3 of 4 characteristic neuroimaging criteria are present. Magnetic resonance imaging (MRI) features that are suggestive of IIH include the presence of (1) an “empty sella”, (2) distension of the optic nerve sheath, (3) transverse sinus stenosis and (4) posterior globe flattening (PGF).

**Table 1:** IIH diagnostic criteria. Table modified according to Friedman et al. [7]

IIH	IIHWOP (IIH without papilledema)	possible IIHWOP
**a.** papilledema	criteria **b-e** fulfilled *plus*:	criteria **b-e** fulfilled *plus*:
**b.** normal neurological examination (except for abducent nerve palsy) **c.** neuroimaging: exclusion of hydrocephalus, mass lesions, meningeal enhancement and cerebral venous or cerebral sinus thrombosis **d.** normal CSF constituents **e.** lumbar puncture opening pressure ≥ 25cmCSF	unilateral or bilateral abducent nerve palsy	presence of 3 of 4 neuroimaging findings: • empty sella turcica • posterior globe flattening • distension of the optic nerve sheath • transverse venous sinus stenosis

PGF describes a flattening of the posterior part of the ocular globe, which has primarily been associated with IIH and with secondary intracranial hypertension. It is considered to result from elevated CSF pressure being transmitted from the subarachnoid space through the optic nerve sheath to the posterior globe [8]. Interestingly, PGF has also been reported in astronauts as a part of spaceflight associated neuro-ocular syndrome [9]. Various studies have found PGF to be among the most specific imaging signs of IIH (range 78–100%, [10–12]), whereas reports on the sensitivity of PGF in IIH vary considerably (44–80%, [10, 13]), possibly resulting from uncertainties in determining the presence of PGF. Currently, the assessment for PGF is based on a subjective radiological review of the available brain MRI. As of today, there are no standardized imaging protocols for the assessment of PGF. Subsequently, PGF is either documented as present or not. Quantitative methods are not routinely utilized. However, the quality of orbital MRI may vary depending on the burden of technical artifacts (e.g. motion artifacts, partial volume-, susceptibility-, or chemical shift artifacts) and on the scanning protocol used for image acquisition (e.g. slice thickness and field strength) [14].

Transorbital ultrasound allows for a non-invasive, dynamic, and high-resolution examination of the posterior aspect of the globe. In IIH, ultrasound is routinely applied for the measurement of the optic nerve sheath diameter (ONSD) as an indicator of elevated intracranial pressure [15]. Additionally, ultrasound has shown utility in the quantitative assessment of papilledema (by measuring the protrusion of the optic nerve head into the globe) and the differentiation of pseudo-papilledema due to the presence of optic disc drusen [16–18]. To the best of our knowledge, ultrasound has not yet been applied to assess PGF in IIH.

This study aimed (1) to evaluate the feasibility of ultrasound imaging of PGF and (2) to develop a quantitative assessment protocol for PGF in IIH and healthy controls using B-mode transorbital ultrasound.

## Methods

This prospective case-control study evaluated PGF on transorbital ultrasound of the globes of IIH patients and matched healthy controls. All subjects gave informed, written consent to participate in this study. Ethical approval was obtained and the study was registered with the German registry for clinical trials (DRKS) (ID-No. DRKS00017815).

### Study population

**Fig. 1:**
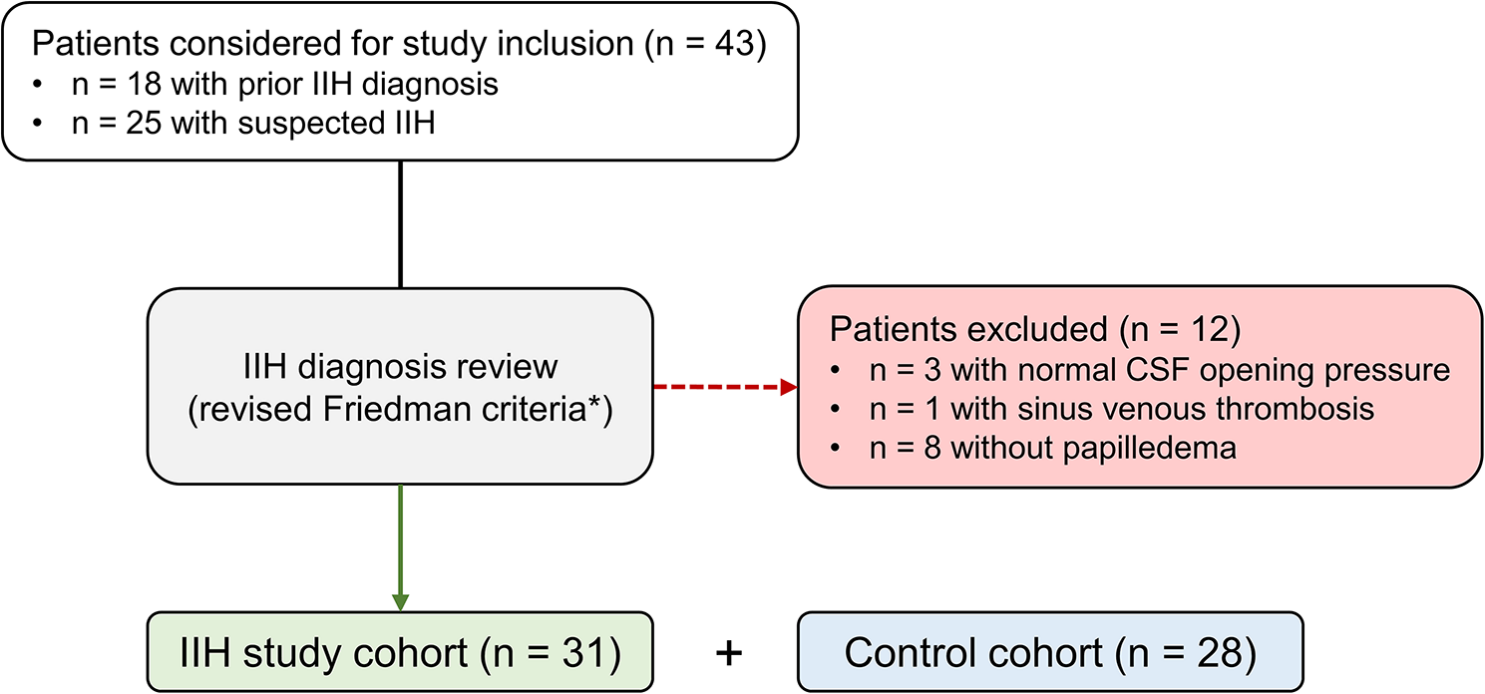
Flow-chart depicting patient selection. Forty-three patients were considered for study inclusion. The IIH diagnosis was reviewed according to the revised Friedman criteria [7]. Patients were excluded from the study if IIH diagnostic criteria were not met and/or a secondary cause of intracranial hypertension was identified

This study included consecutive adult patients who underwent diagnostic evaluation or inpatient treatment for IIH between January 2021 and November 2022 at our tertiary care neurological department. Patients were considered for study inclusion if (1) a diagnosis of IIH had been established prior to current admission or (2) a diagnosis of IIH was suspected. Consecutively, diagnosis of IIH was reviewed by the study team according to the revised Friedman criteria [7], based on ophthalmological and neurological findings, cerebral magnetic resonance imaging and measurements of cerebrospinal fluid opening pressure. Patients were excluded from the study if IIH diagnostic criteria were not met and/or a secondary cause of intracranial hypertension was identified. Patients without fundoscopic evidence of papilledema (current or in medical history) were not included in this study. Figure 1 depicts the patient selection process.

Healthy subjects, free of current or past neurological or ophthalmological disorders, matched for age and sex were recruited as controls between January 2021 and February 2023.

### Transorbital ultrasound

Ultrasound examinations were performed by two experienced investigators (LD and TK) using a 7.5-MHz linear-array transducer of the Aplio 300 ultrasound system (Canon Medical Systems Corp., Ōtawara, Tochigi, Japan) and an orbital imaging preset, limiting the acoustic output exposure levels according to the recommendations of the U.S. Food and Drug Administration [19]. Both investigators were aware of the participants medical records and IIH diagnosis at the time of the examination. Care was taken to limit the ultrasound exposure (dwell time) to less than a minute for each eye. Standard B-mode transorbital ultrasound in the axial plane included measurements of optic nerve diameter (OND), optic nerve sheath diameter (ONSD) and optic disc size. If ONSD and optic disc size differed between a patients’ eyes, the most severely affected eye (i.e. the higher value) was used for subsequent group comparison. OND values are reported as the mean value of both eyes.

**Fig. 2:**
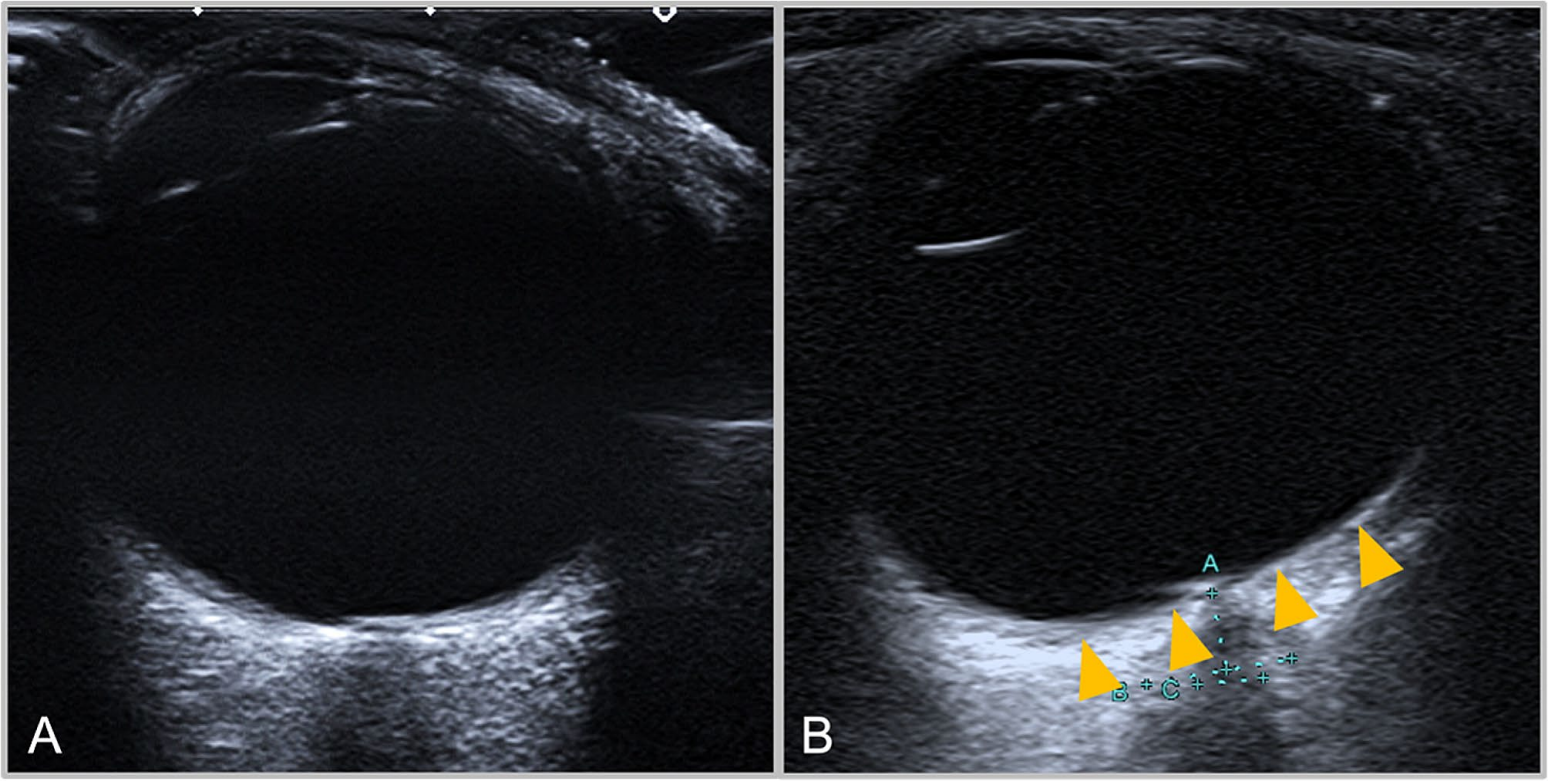
Two B-mode transorbital sonographic images of two individual globes in the axial plane. Image A shows a normal globe. Image B depicts posterior globe flattening in a globe with straightening of the outward convexity of the sclera at the area of attachment to the optic nerve (yellow arrowheads)

For the qualitative assessment of the presence of PGF, the acquired B-mode images of each orbit were reviewed by TK and LD. Posterior globe flattening was considered present if there was a straightening of the normal outward convexity of the sclera at the area of attachment to the optic nerve in at least one eye (Fig. 2).

**Fig. 3:**
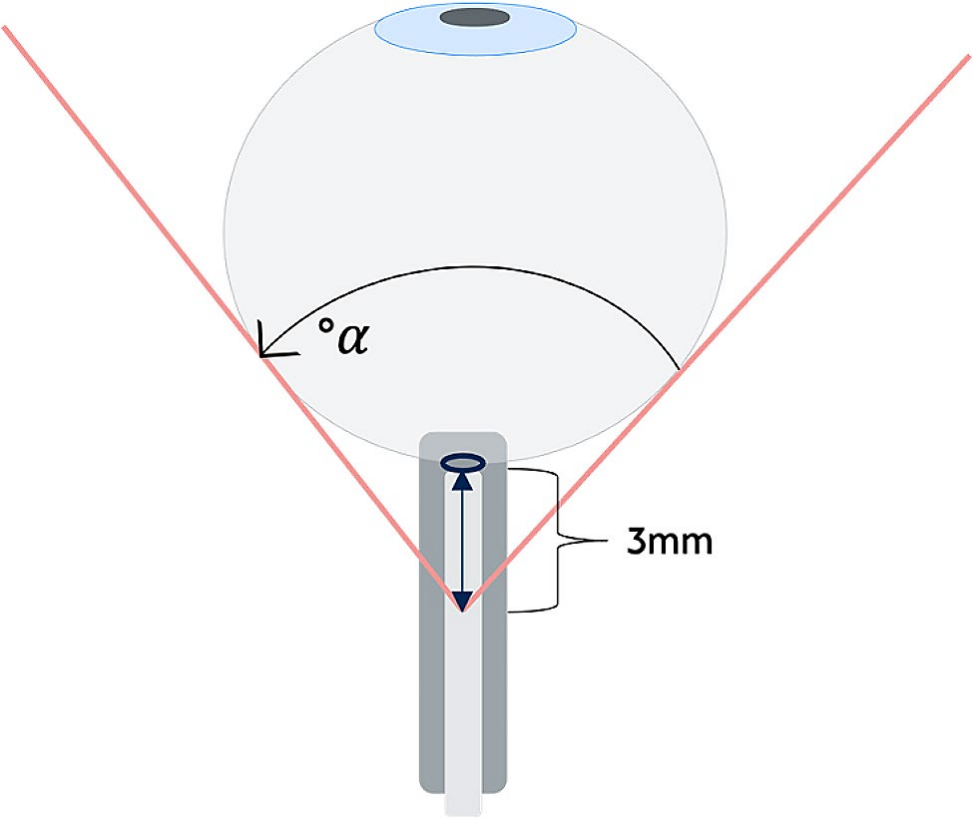
Graphic illustration of the PGA (α) measured at 3.0 mm distance from the ophthalmic lamina cribrosa

**Fig. 4:**
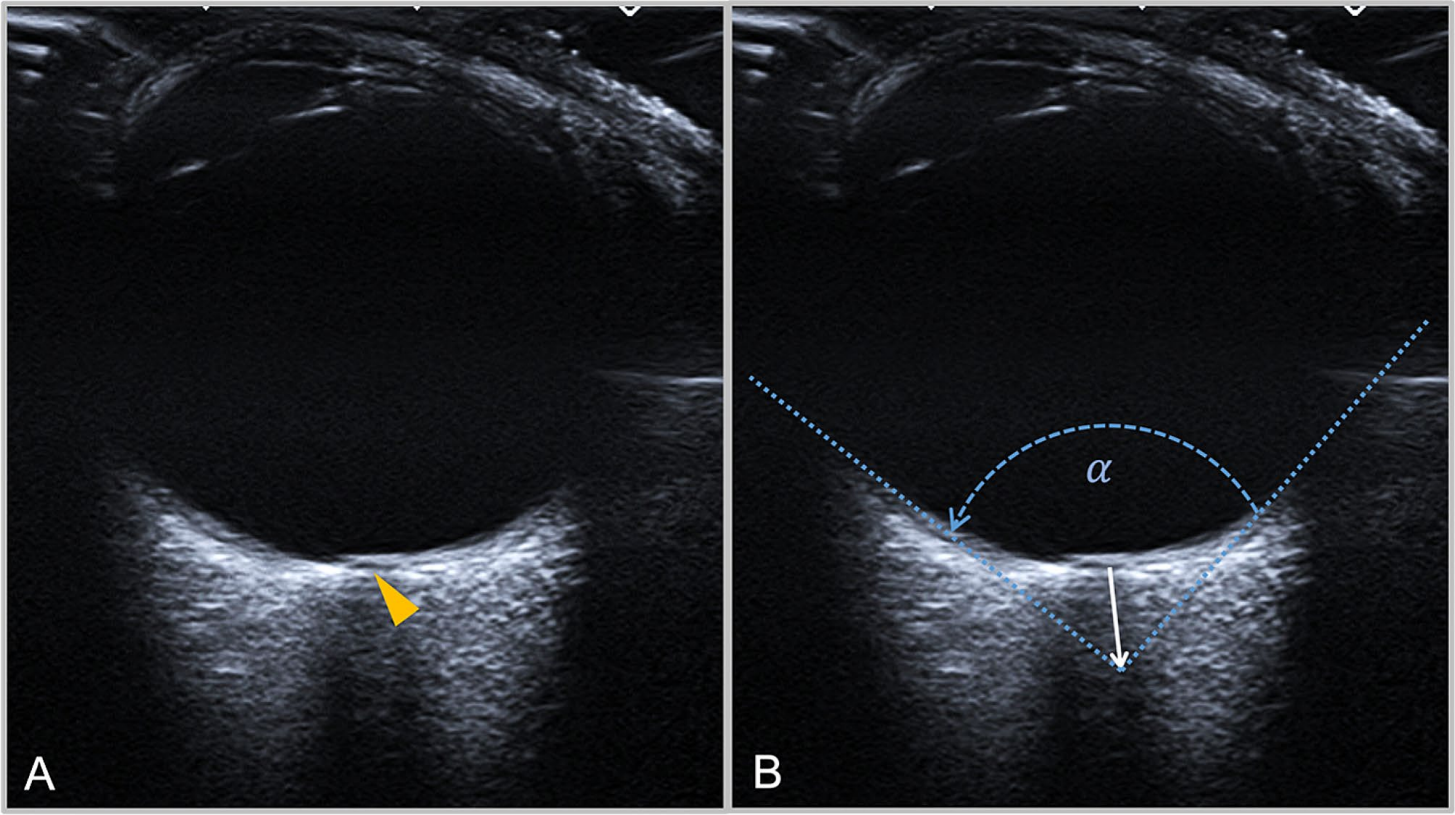
Transorbital ultrasound of one globe in the axial plane. Picture A shows the original image, the yellow arrowhead marks the ophthalmic lamina cribrosa. Picture B shows exemplary measurement of the PGA at 3.0 mm distance from the ophthalmic lamina cribrosa

**Fig. 5:**
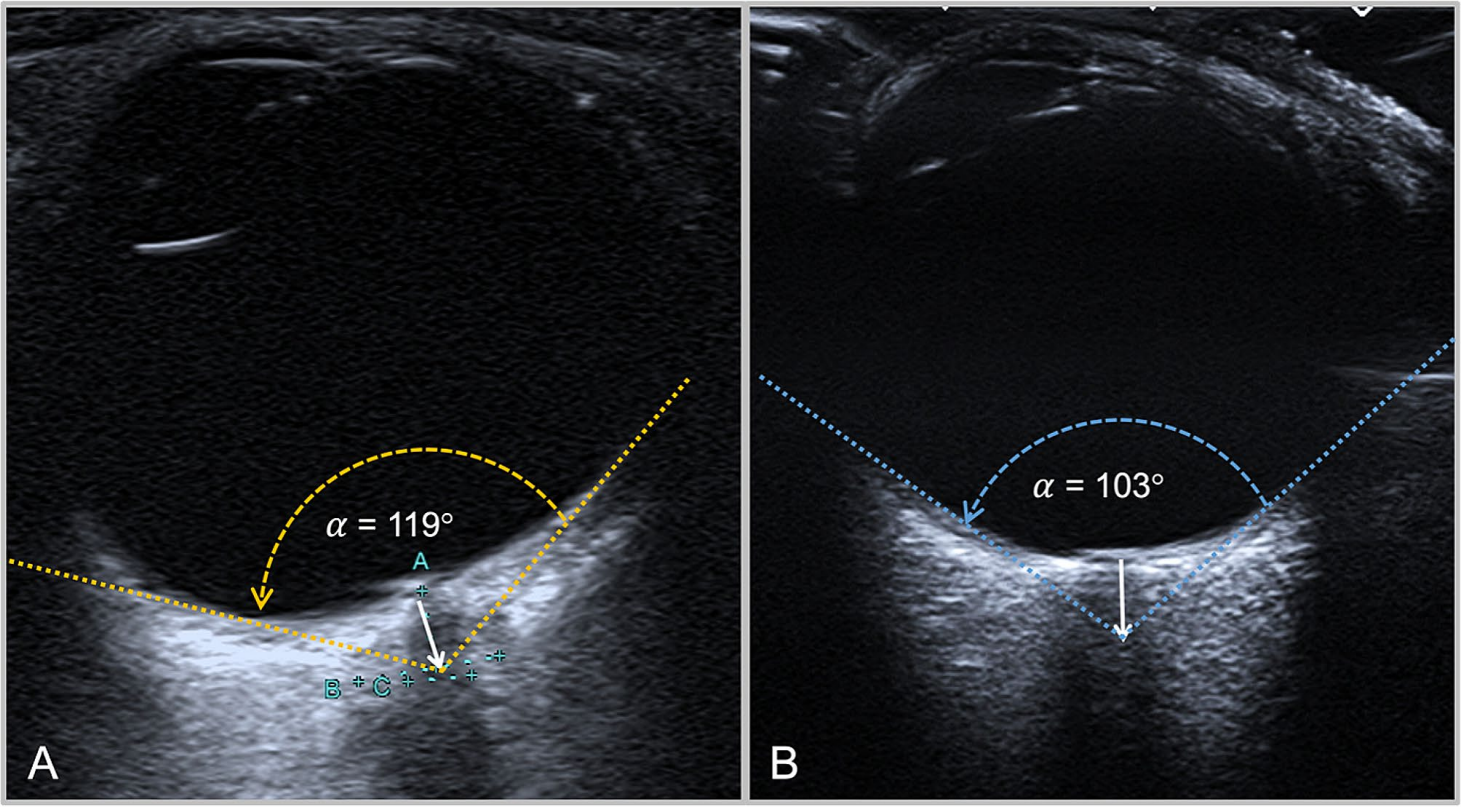
Transorbital ultrasound images of one globe in the axial plane depicting measurements of the PGA at 3.0 mm distance from the ophthalmic lamina cribrosa in a globe with PGF (**A**) and a normal globe (**B**)

For the quantitative assessment of PGF, a transverse imaging plane showing the insertion of the optic nerve into the eyeball was set first. Next, the vertex for angular measurement was marked at the center of the optic nerve at a predefined distance from the lamina cribrosa, at 2.0 mm, 2.5 mm and 3.0 mm, respectively. Starting from the vertex, the two angle legs were tangentially aligned with the medial and lateral border of the anechoic vitreous body. Subsequently, the *Posterior globe angle* (PGA = α) was measured between the angle legs (Figs. 3, 4 and 5). If the PGA differed between a patients’ eyes, the most severely affected eye (i.e. the higher value) was used for statistical analysis.

**Fig. 6:**
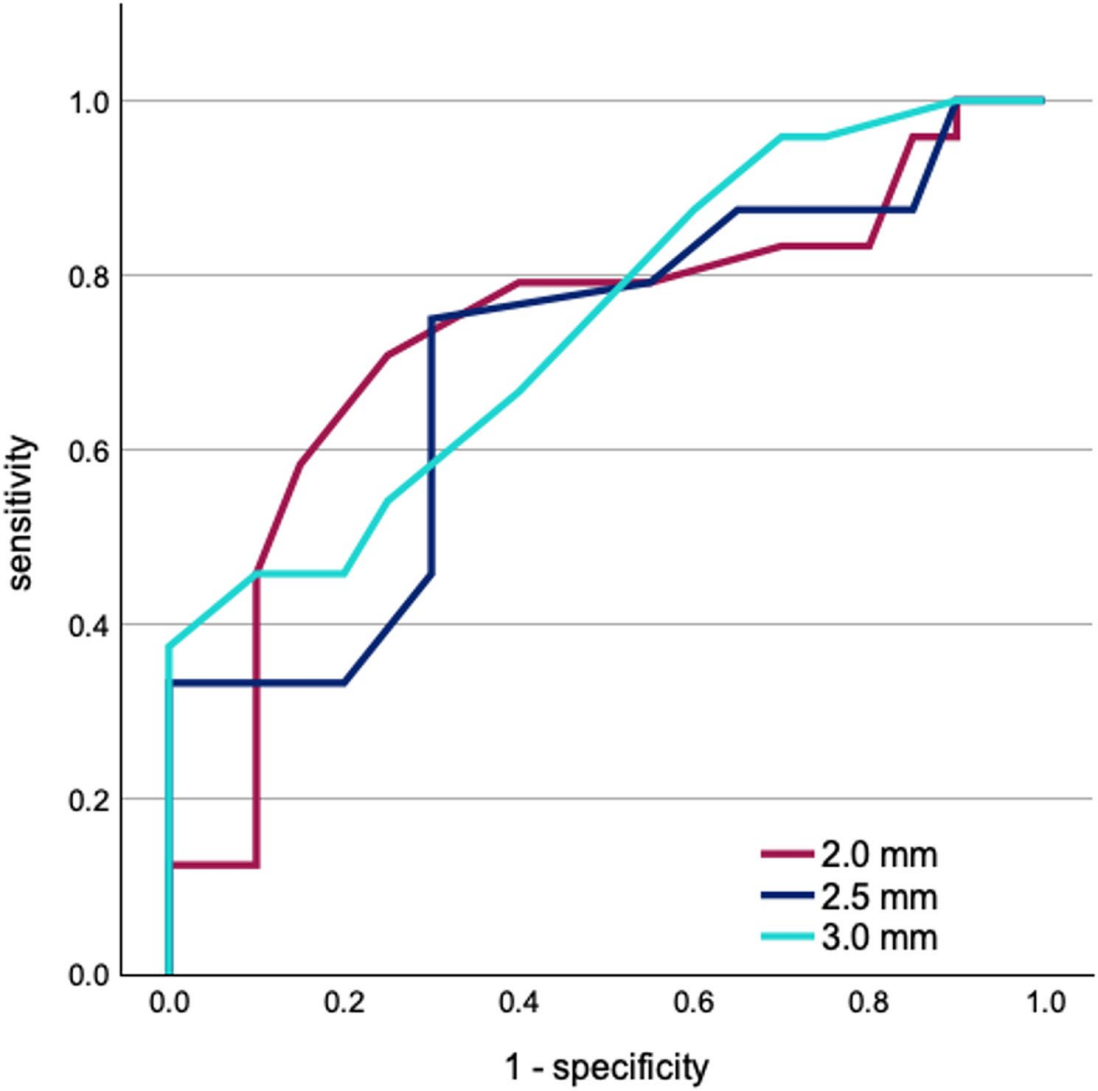
ROC curves of the PGA values obtained by transorbital ultrasound to differentiate the IIH patients from healthy controls. PGA was measured at 3.0 mm, 2.5 mm and 2.0 mm distance from the ophthalmic lamina cribrosa. Corresponding data is summarized in Table 4

### Statistical analysis

Statistical analyses were performed using IBM SPSS Statistics software (IBM SPSS Statistics, Version 29.0. Armonk, NY: IBM Corp.). The continuous variables are presented as mean ± standard deviation.

Given that the Q-Q plots and histograms indicated normal distribution for all continuous variables, two-tailed t-tests were employed for comparing groups across these variables. Two-sided 95% confidence intervals are provided for the differences in means. *p*-values were adjusted for multiple comparisons using the Bonferroni-Holm procedure, where applicable. Group comparisons concerning sex were computed using Fisher’s exact test. Sensitivity and specificity were calculated by utilizing receiver operating characteristic (ROC) curves and the Youden index for cut-point estimation.

## Results

### Study population

During the study period 43 patients were considered for study inclusion. Among them, 25 patients were referred with suspected IIH, while 18 patients had been diagnosed with IIH prior to current admission. Twelve patients did not meet the diagnostic criteria for IIH and were excluded for either (1) lack of papilledema (*n* = 8), and/or (2) lack of elevated CSF opening pressure (*n* = 3) or (3) due to the identification of sinus venous thrombosis (*n* = 1).

The final study cohort consisted of 31 consecutive IIH patients (29 female; 37.3 ± 12.3 years) and 28 healthy controls (21 female; 33.3 ± 11.8 years). Patients and controls did not significantly differ regarding age (*p* = 0.200) and sex (*p* = 0.071). The IIH cohort included patients with active IIH as determined by presence of papilledema, and patients in ocular remission (i.e. no current evidence of papilledema). When they participated in study, 65% (20/31) of the IIH cohort had papilledema, 13% (4/31) had optic nerve atrophy and 23% (7/31) had no evidence of papilledema anymore. The mean CSF opening pressure before measurement of PGF was 36 ± 10.5 cmCSF (range: 17–55 cmCSF). Table 2 shows the characteristics of the study population.

**Table 2:** Characteristics of the study population

	IIH patients *n* = 31	controls *n* = 28	*p*
female male	29 (93.5%) 2 (6.5%)	21 (75%) 7 (25%)	0.071
age (years)	37.3 ± 12.3	33.3 ± 11.8	0.200
papilledema		-	-
- yes - no optic atrophy	20 (64.5%) 4 (12.9%) 7 (22.6%)		

### Transorbital ultrasound

Table 3 summarizes transorbital ultrasound measurements in IIH patients and controls. OND (2.6 ± 0.5 mm vs. 2.9 ± 0.5 mm, *p* = 0.031) and ONSD (6.0 ± 0.7 mm vs. 5.1 ± 0.5 mm, *p* < 0.001) significantly differed between IIH patients and controls. No significant differences were observed for optic disc measurements (patients: 0.7 ± 0.5 mm, controls: 0.5 ± 0.9 mm, *p* = 0.419, 95% CI: -0.6–0.3 mm).

**Table 3:** Results of the transorbital ultrasound. Values are presented as mean ± standard deviation. *Bonferroni-Holm corrected values

	IIH patients *n* = 31	controls *n* = 28	*p*	95% CI
**OND** [mm]	2.6 ± 0.5	2.9 ± 0.5	0.031	0.03–0.6
**ONSD** [mm]	6.0 ± 0.7	5.1 ± 0.5	< 0.001	0.5–1.3
**optic disc** [mm]	0.7 ± 0.5	0.5 ± 0.9	0.419	-0.6–0.3
**PGA (α)°** α at 3.0 mm	116.5 ± 5.5	111.7 ± 2.9	< 0.001*	2.5–7.2
α at 2.5 mm	120.7 ± 5.4	116.9 ± 3.1	0.016*	1.0–6.6
α at 2.0 mm	126.0 ± 4.5	122.8 ± 3.3	0.016*	0.8–5.7

Abbreviations: OND = optic nerve diameter, ONSD = optic nerve sheath diameter, PGA = posterior globe angle

Qualitative assessment of the globes found PGF to be present on transorbital ultrasound in 39% of IIH patients. None of the control subjects exhibited PGF. The PGA measured at 3.0 mm, 2.5 mm and 2.0 mm distance from the lamina cribrosa all significantly differed between IIH patients and the control group (Table 3). The PGA at 3.0 mm showed the highest difference of the means between IIH patients and controls (95% CI: 2.5–7.2°, *p* < 0.001). Similarly, in ROC analysis the PGA at 3.0 mm yielded the highest area under the curve (AUC = 0.74) when compared with PGA measured at 2.5 mm and 2.0 mm distance from the lamina cribrosa (Table 4, Fig. 6). A predefined cutoff ≥ 118.5° PGA_3mm_ identified IIH patients with a specificity of 100%, retaining a sensitivity of 37,5% (Youden index: 0.375). Table 4 summarizes sensitivity and specificity obtained by ROC analysis and optimal cutoff points estimated by Youden index for PGA measurements at 3.0 mm, 2.5 mm and 2.0 mm distance from the lamina cribrosa.

**Table 4:** Sensitivity and specificity analysis for the PGA measured at 3.0 mm, 2.5 mm and 2.0 mm distance from the lamina cribrosa. Area under the curve (AUC), sensitivity, and specificity were obtained via ROC analysis. ROC curves are depicted in Fig. 6. Optimal cutoff points were estimated using the Youden index. The maximum value of the Youden index is 1 (perfect test) and the minimum is 0

Distance	Area under the curve	Cut-off PGA (α)°	Sensitivity (%)	Specificity (%)	Youden index
**3.0 mm**	0.744	≥ 118.5	37.5	100	0.375
**2.5 mm**	0.704	≥ 117.5	75.0	70.0	0.45
**2.0 mm**	0.732	≥ 124.5	71.0	75.0	0.46

## Discussion

This study applied transorbital ultrasound to quantify PGF in patients with IIH using the *posterior globe angle (PGA)*. Here, we were able to show for the first time, that PGA measurements differ significantly between IIH patients and healthy controls. We compared PGA measurements at different positions of the angle vertex using ROC analysis, which found the highest AUC for measurements at 3.0 mm distance from the lamina cribrosa (AUC = 0.74). A cutoff of ≥ 118.5° PGA would identify IIH patients with 100% specificity while retaining a sensitivity of 37.5%. The results of this preliminary case-control study indicate that transorbital ultrasound may be applied to identify and quantify PGF in IIH patients.

In 1993, Gibby et al. described a “flattening of [the] normal outward convexity” of the posterior sclera on computed tomography images of IIH patients [20]. Since then, multiple studies have investigated PGF in IIH using magnetic resonance imaging [10, 11, 13, 21–26]. The overwhelming majority of these studies based the radiological assessment on a qualitative, subjective evaluation of axial T2-weighted images. Consequently, reports on diagnostic accuracy have produced inconsistent results, with sensitivity and specificity of PGF in IIH varying between 28.8 and 78.6% and 59.5–99.1%, respectively [27]. Similarly, reports by Delen et al. and Alperin et al. only show moderate interrater-reliability of quantitative PGF evaluation (k = 0.46–0.64) [22, 28]. Still, the available studies suggest that PGF may show diagnostic utility as a rather specific imaging finding.

In 2013, Alperin et al. reported the first quantitative assessment of PGF and optic nerve protrusion in IIH using MRI in a cohort of 7 IIH patients and 6 healthy controls [28]. The authors developed software to convert the three-dimensional structure of the globe into a color-coded two-dimensional distance map, visualizing the distances between the center of the globe to different points on the posterior sclera. While Alperin et al. argued automated, quantitative measurements would likely improve the detection of globe distortions in IIH, their findings have yet to be confirmed in subsequent studies using larger cohorts.

Compared to MRI, transorbital ultrasound is a cost-effective, widely available, and easy-to-use examination method that is particularly suitable for follow-up examinations. Since it is already established to measure OND and papilledema in routine clinical practice, it seems reasonable to extend its scope of application to assess deformations of the posterior ocular globe in IIH. The diagnosis of IIH remains a challenge, as reports on diagnostic error are prominent in the medical literature [6, 29]. The improvement of available diagnostic modalities, as well as the establishment of new diagnostic approaches could improve detection and disease monitoring of IIH.

This preliminary study is limited by its small case size due to the rarity of the disease. Although the current diagnostic criteria were applied, the IIH cohort included patients with active IIH and patients in the stage of ocular remission, which may have affected our results. In addition, the examiners who performed the ultrasound were not blinded to the patients’ medical history, including IIH diagnosis, which introduces a risk of bias. Consequently, further studies are necessary to confirm our findings using extended cohorts, a blinded study design and concomitant MR-imaging of the ocular globes as reference.

It is currently unclear to what extent PGA measurements are influenced by morphological variations of the ocular globe, e.g. in myopia or hyperopia. Theoretically, transorbital ultrasound examination could be extended to include further measurements to adjust for differences in ocular globe morphology. For PGA measurements, a transverse image plane at the insertion of the optic nerve into the eyeball is set. However, bulb movements may impede the assessment and lead to oblique imaging planes, possibly distorting measurements. We recommend placing the examiner’s proximal wrist on the patient’s forehead to avoid unnecessary pressure on the eye during the assessment and to ensure sufficient application of ultrasound gel. These precautions, in addition to direct instruction of the patient, help to reduce the occurrence of bulbar movements.

## Conclusion

To conclude, this prospective case-control study is the first to apply transorbital ultrasound to depict and to quantify PGF in IIH. PGA measurements differed significantly between IIH patients and controls. Measured at 3.0 mm distance from the lamina cribrosa, a predefined PGA cut-off of ≥ 118.5° yielded a sensitivity of 37.5% and specificity of 100% to distinguish IIH patients from controls. However, given the limitations of this study, its results should be considered preliminary. Prospective, multicenter studies with extended cohorts and blinded design are needed to confirm the diagnostic utility and reproducibility of transorbital ultrasound for the assessment of PGF in IIH.

## Data Availability

The datasets generated for the current study are not publicly available but are available from the corresponding author on reasonable request.

## Abbreviations

IIH: Idiopathic intracranial hypertension
PGF: Posterior globe flattening
PGA: Posterior globe angle
MRI: Magnetic resonance imaging
OND: Optic nerve diameter
ONSD: Optic nerve sheath diameter
CSF: Cerebrospinal fluid
ROC: Receiver operating characteristic
AUC: Area under the curve
